# The Clinical Features and Prognosis of Idiopathic and Infection-Triggered Acute Exacerbation of Idiopathic Inflammatory Myopathy-Associated Interstitial Lung Disease: A Preliminary Study

**DOI:** 10.3390/diagnostics15192516

**Published:** 2025-10-03

**Authors:** Jingping Zhang, Kai Yang, Lingfei Mo, Liyu He, Jiayin Tong, He Hei, Yuting Zhang, Yadan Sheng, Blessed Kondowe, Chenwang Jin

**Affiliations:** 1 Department of Medical Imaging, The First Affiliated Hospital of Xi’an Jiaotong University, 277 West Yanta Road, Xi’an 710061, China; zhangjp@xjtufh.edu.cn (J.Z.);; 2 Department of Rheumatology, The First Affiliated Hospital of Xi’an Jiaotong University, 277 West Yanta Road, Xi’an 710061, China; 3 Department of Radiology, Mzuzu Central Hospital, Private Bag 209, Mzuzu, Malawi; 4 Shaanxi Engineering Research Center of Computational Imaging and Medical Intelligence, 277 West Yanta Road, Xi’an 710061, China

**Keywords:** acute exacerbation, infection, interstitial lung disease, idiopathic inflammatory myopathies, mortality

## Abstract

**Background**: Acute exacerbation (AE) of idiopathic inflammatory myopathy-associated interstitial lung disease (IIM-ILD) is fatal. Infection is one of the most important triggers of the AE of IIM-ILD. We evaluated the clinical features and prognosis of idiopathic (I-AE) and infection-triggered (iT-AE) acute exacerbation in IIM-ILD patients. **Methods**: We retrospectively reviewed 278 consecutive patients with IIM admitted to our hospital between January 2014 and December 2020. Among them, 69 patients experienced AE of IIM-ILD, including 34 with I-AE and 35 with iT-AE. Clinical features and short- and long-term outcomes were analyzed in this preliminary study. **Results**: Compared with I-AE, patients with iT-AE presented with lower hemoglobin and PaO_2_/FiO_2_ ratios but higher pulse, body temperature, white blood cell count, neutrophil percentage (NEU), C-reactive protein, erythrocyte sedimentation rates, lactate dehydrogenase, and hydroxybutyrate dehydrogenase levels. They also had more extensive ground-glass opacities (GGOs) on high-resolution computed tomography (all *p* < 0.05). Mortality was significantly higher in iT-AE than that in I-AE at 30 days (28.6% vs. 5.9%), 90 days (34.3% vs. 14.9%), and 1 year (54.3% vs. 17.6%; log-rank test, *p* = 0.002). Multivariate logistic regression showed that the combination of NEU and GGO extent could help discriminate iT-AE from I-AE (area under the receiver operating characteristic curve: 0.812; 95% confidence interval: 0.711–0.913; sensitivity: 71.4%, specificity: 73.5%, accuracy: 72.5%). **Conclusion**: This study found that iT-AE patients exhibited more severe hyperinflammation and markedly worse survival than I-AE patients. Combining NEU and GGO extent may assist in differentiating AE subtypes. Larger prospective studies are required to validate these findings.

## 1. Introduction

Interstitial lung disease (ILD) is one of the most common extra-muscular complications of idiopathic inflammatory myopathies (IIMs), with a prevalence ranging from 19.9% to 42.6% depending on the study series and the definition used [1]. It is the primary cause of death and hospitalization in patients with IIMs, with a reported mortality rate ranging from 7.5% to 44% [1]. Acute exacerbation (AE) is a catastrophic event in patients with ILD that can occur anytime and is associated with significant morbidity and mortality [2,3,4]. According to a recent systematic review study, the frequency of AE in rheumatic-disease-associated ILD (RD-ILD) ranged from 4.3 to 32.9%, with the incidence rate being 3.19 and 5.77 per 100 patient-years, and all-cause mortality was between 30.0 and 58.3% at 90 days; rheumatoid arthritis and polymyositis/dermatomyositis (PM/DM) were the two main underlying rheumatic diseases [3].

The definition of AE for RD-ILD applied in most previous studies was based on the criteria indicated for AE of idiopathic pulmonary fibrosis (AE-IPF), established in 2007 and updated in 2016 [5,6]. Recently, Luppi, F. et al. proposed a specific definition for AE of RD-ILD based on its characteristics with consideration of the 2016 definition of AE-IPF [4]. In this document, AE of RD-ILD is defined as acute, clinically significant respiratory worsening typically within one month, characterized by a new widespread alveolar abnormality on high-resolution computed tomography (HRCT) superimposed onto a background pattern of RD-ILD [4]. Like the 2016 definition of AE-IPF, this diagnostic criterion also emphasizes the discrimination between a triggered AE (e.g., infection, anti-rheumatic drug use, microaspiration, surgery, and air pollution) and an idiopathic AE, where no trigger can be identified [4,6]. This further differentiation highlights the need to comprehensively understand the pathogenesis of AE-RD-ILD and develop more precise treatments to enhance patient outcomes. Among all the triggers, pulmonary infection undoubtedly stands out as the most common and important one. The pivotal role of infection in AE-IPF has been extensively documented, and infection-triggered AE-IPF has been recognized and studied as a distinct phenotype from idiopathic AE-IPF in many studies [7,8,9,10]. It is reported that infection-triggered AE-IPF has distinct clinical characteristics and carries a significantly higher risk of mortality compared to that for idiopathic AE-IPF [9,10]. In recent years, there has been a significant surge in research pertaining to AE of RD-ILD, encompassing its clinical characteristics, risk factors, and prognosis and a comparative analysis with AE-IPF [3,11,12,13,14]. However, limited data are available regarding clinical characteristics and prognosis according to the etiologies of AE of RD-ILD. Lee et al. investigated the treatment outcomes for 36 patients with AE of myositis-related ILD by etiology and reported that infectious AE is a significant cause of mortality [15]. However, they failed to distinguish infectious AE from non-infectious AE based on a small sample. In clinical practice, distinguishing between infectious and non-infectious causes of AE in patients with IIM-ILD is crucial for individualized treatment decision-making [7]. This study aimed to investigate the differences in clinical characteristics and prognosis between idiopathic AE (I-AE) and infection-triggered AE (iT-AE) in patients with IIM-ILD and to identify potential early indicators that may facilitate differentiation between these subtypes and inform clinical management.

## 2. Materials and Methods

### 2.1. Study Population

This retrospective study was performed in line with the principles of the Declaration of Helsinki and was reviewed and approved by the Human Ethics Committee of our hospital [approval number: XJTU1AF2024LSYY-268; approval date: 12 August 2024]. Written informed consent was waived by the ethics committee due to the study’s retrospective nature. Clinical and imaging data was anonymized.

**Figure 1:**
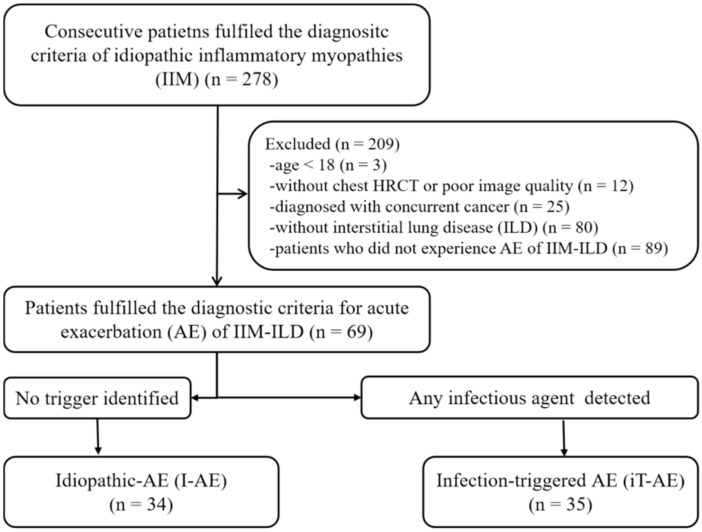
Flowchart of eligible patients. AE, acute exacerbation; ILD, interstitial lung disease.

We retrospectively reviewed the medical records of 278 consecutive patients with IIMs who were admitted to the rheumatology ward of our hospital between January 2014 and December 2020 to establish our study cohort. IIMs were defined using previous diagnostic criteria [16,17]. ILD was diagnosed based on the HRCT findings following the ATS/ERS criteria [18,19,20]. We defined AE of IIM-ILD according to the previously proposed criteria by Fabrizio Luppi et al. for rheumatic disease as (1) a previous or concurrent diagnosis of ILD; (2) acute worsening or development of dyspnea <1 month; (3) new bilateral ground-glass opacity and/or consolidation superimposed onto a background pattern of ILD on HRCT; and (4) deterioration not fully explained by cardiac failure, fluid overload, or disease-modifying anti-rheumatic drug use [4]. The investigators reassessed both the diagnoses of IIM-ILD and AE and determined them based on their consensus. Patients meeting the diagnostic criteria with an available chest HRCT at AE onset were included in this study. The exclusion criteria were as follows: (1) patients who were under 18 years of age, (2) had no chest HRCT scan or one with poor image quality, (3) without ILD, and (4) diagnosed with concurrent cancer. As a result, 69 consecutive patients with AE of IIM-ILD were ultimately included in this study. Furthermore, patients were categorized as having experienced either an idiopathic AE (I-AE) when no trigger was identified after comprehensive diagnostic work-up or an infection-triggered AE (iT-AE) when an infectious etiology was confirmed [4] (Figure 1). All patients underwent blood tests, sputum/blood cultures, and bronchoalveolar lavage (BAL) when clinically feasible, within 48 h of admission. Pathogen detection included conventional cultures, polymerase chain reaction (PCR) assays for respiratory viruses, and serum fungal markers (β-D-glucan, galactomannan). iT-AE was defined as microbiological or virological confirmation of bacteria, viruses, or fungi from these samples alongside consistent clinical–radiological features. Cases with mixed or uncertain findings were classified as iT-AE if any infectious agent was detected [15,21]. Patients without pathogen detection were designated as I-AE only after excluding alternative triggers (e.g., aspiration, drug-induced toxicity, environmental exposures) through a comprehensive evaluation of clinical history, imaging, and treatment records. All patients received pre-AE immunosuppressive maintenance therapy, with iT-AE cases receiving pathogen-directed antimicrobial treatment.

### 2.2. Data Collection

Clinical data were collected from the Biobank of the First Affiliated Hospital of Xi’an Jiaotong University, including demographics [age, sex, body mass index (BMI), systolic blood pressure (SBP), diastolic blood pressure (DBP), smoking status] and laboratory findings [white blood cell (WBC) count, platelets (PLTs), hemoglobin (HGB), neutrophil percentage (NEU), C-reactive protein (CRP), erythrocyte sedimentation rate (ESR), creatine kinase (CK), lactate dehydrogenase (LDH), hydroxybutyrate dehydrogenase (HBDH), and PaO_2_/FiO_2_ ratio]. Pulse and body temperature at admission, IIM subtype, disease duration of IIMs, IIM-associated and specific autoantibodies (available for 48 patients), comorbidities (e.g., diabetes mellitus, hypertension, coronary heart disease, etc.), and treatment regimens before and after AE onset were retrieved from the medical records. The primary outcome was 1-year all-cause mortality following AE onset, while secondary outcomes included 30-day and 90-day mortality. Survival status was determined by reviewing medical records and/or confirmed via telephone interviews. Patients were followed from AE admission until death or the end of the follow-up period on 31 December 2021. Patients who were alive at the last follow-up or lost to follow-up were censored at the date of last known contact. Four patients were lost to follow-up in the 2nd, 4th, 7th, and 10th months after AE onset, respectively, and were censored at those time points. This study re-analyzes part of the data from our previously published study [22].

### 2.3. Evaluation of HRCT Characteristics

The HRCT examinations were performed within 7 days of symptom onset (median: 0 days; range: 0–7 days) during acute exacerbation episodes, using scanners from multiple vendors: Philips Healthcare (Best, The Netherlands), Toshiba Medical Systems (Otawara, Japan), United Imaging Healthcare (Shanghai, China), and GE Healthcare (Chicago, IL, USA). All patients underwent HRCT scans at the end-inspiration phase in the supine position. Scanning protocol: Tube voltage: 120 kV; automatic tube current; a reconstruction matrix of either 512 × 512 or 1024 × 1024; and a scanning range from the apex of the lung to the costophrenic angle. HRCT images were anonymized and reviewed by two expert thoracic radiologists with 12 years and 24 years of experience independently with window settings appropriate for viewing the lung parenchyma (window width: 1500–1600 HU; window level: −500 to −600 HU) in a random order without knowledge of any of the clinical information. The extent of parenchymal abnormalities on HRCT was evaluated according to the Fleischner Society’s glossary of terms for thoracic imaging and Walsh’s scoring system for RD-ILD [23,24]. Briefly, the extent of five parenchymal abnormalities on HRCT, namely ground-glass opacities (GGOs), reticulation, honeycombing, consolidation, and emphysema, was scored to the nearest 5% at each anatomical level at six levels: the aortic arch; 1 cm below the level of the carina; the right pulmonary venous confluence; the midpoint between level 3 and level 5; 1 cm above the dome of the right hemidiaphragm; and 2 cm below the dome of the right hemidiaphragm. The results for each of these six levels were then averaged.

### 2.4. Statistical Analyses

All statistical analyses were performed using R 4.1.0 (R Core Team, R: A Language and Environment for Statistical Computing 2013, available at http://www.rproject.org/, accessed on 31 March 2021). The Shapiro–Wilk normality test tested the normality of continuous variables. Continuous variables were expressed as means with standard deviations (SD) if normally distributed and medians with interquartile ranges (M, IQR) if skewed. Unordered categorical variables were given as numbers and percentages. Student’s *t*-test or the Mann–Whitney *U* test was applied to comparing continuous variables. *X*^2^ or Fisher’s exact test was used to compare categorical variables. All tests were two-sided, and *p* ˂ 0.05 was considered statistically significant. The intraclass correlation coefficient (ICC) of the absolute agreement based on a two-way random model was used to calculate the interobserver agreement of the extent of HRCT findings [25]. An ICC over 0.81 indicated excellent test–retest reliability. The interobserver correlations were assessed using Spearman’s rank correlation test (r), and an r greater than 0.7 corresponded to excellent reliability (Appendix A Table A1).

Variables considered clinically relevant or with *p* ˂ 0.05 in the univariate logistic analysis were entered into a multivariate logistic regression model. Multicollinearity between variables was checked by calculating the variance inflation factor (VIF), and variables were removed if the VIF was >10. Receiver operating characteristic (ROC) curves were generated for combined or individual parameters to assess the area under the ROC curve (AUC) for differentiating I-AE and iT-AE and obtaining the cut-off threshold values corresponding to the highest value of the Jordan index. The survival analysis was performed using Kaplan–Meier curves, and group differences were assessed using the log-rank test.

## 3. Results

### 3.1. Characteristics at AE Onset

The clinical characteristics of I-AE and iT-AE of IIM-ILD are shown in Table 1. The mean age at AE onset for IIM-ILD patients was 50.7 (10.0) years, and 79.7% were women. Of the 69 cases, 34 (49.3%) were I-AE cases, and 35 (50.7%) were iT-AE cases. There is no sex predominance or age gap between I-AE and iT-AE patients. Among the 35 iT-AE patients, 9 patients were infected by a virus (5 by Epstein–Barr virus, 1 by cytomegalovirus, 1 by respiratory syncytial virus, 1 by Influenza B virus, 1 by Influenza A virus); 1 patient was infected by mycoplasma pneumoniae; 8 patients were infected by a fungus (4 by Candida albicans, 1 by Aspergillus fumigatus, 3 by Pneumocystis jirovecii), 5 patients were infected by bacteria (1 by Pseudomonas aeruginosa, 1 by Staphylococcus aureus, 3 by Legionella pneumophila); 12 patients had a mixed infection.

There are no significant differences in blood pressure, body mass index, IIM subtypes, autoantibody, IIM disease duration, smoking history, and comorbidity between I-AE patients and iT-AE patients. I-AE patients show lower pulse, body temperature, NEU, and CRP, ESR, LDH, HBDH levels and higher HGB levels and PaO_2_/FiO_2_ ratios than those in iT-AE patients (*p* < 0.05). The WBC count is slightly higher in iT-AE patients than that in I-AE patients, with borderline statistical significance (*p* = 0.071). Other laboratory findings, including PLTs and CK, show no intergroup difference.

The treatment regimens before AE onset were mainly maintenance immunosuppressive therapy, and there was no significant difference between the two groups (*p* > 0.05). The treatment regimens after AE are slightly different between the two groups. More I-AE patients received cyclophosphamide than iT-AE patients (*p* = 0.05).

Regarding radiological characteristics, I-AE patients show a lower extent of GGO on HRCT than iT-AE patients (*p* < 0.05); the other characteristics show no significant intergroup differences.

### 3.2. Mortality

**Figure 2:**
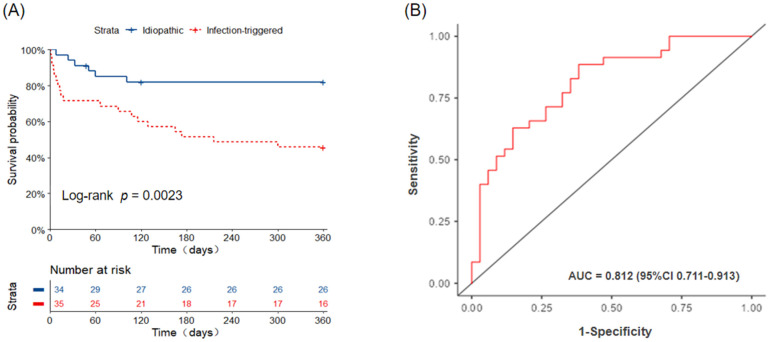
(**A**) Kaplan–Meier curve. Survival after AE, idiopathic-AE versus infection-triggered AE. AE, acute exacerbation. (**B**) ROC curve for differentiating idiopathic and infection-triggered AE of IIM-ILD. AE, acute exacerbation; IIM-ILD, idiopathic inflammatory myopathy-associated interstitial lung disease.

Six patients in the I-AE group (one due to sudden cardiac arrest and five due to progressive respiratory failure) and nineteen patients in the iT-AE group (one due to sudden cardiac arrest, one due to an acute pulmonary embolism, and seventeen due to progressive respiratory failure) died within 1 year of AE onset. The 30-day, 90-day, and 1-year all-cause mortality rates were 5.9%, 14.9%, and 17.6% in the I-AE group and 28.6%, 34.3%, and 54.3% in the iT-AE group, respectively. Mortality was significantly higher in patients with iT-AE compared with those with I-AE (log-rank test, *p* = 0.002). The Kaplan–Meier survival analysis confirmed a significant difference in 1-year cumulative survival probability between the two groups (Figure 2A).

### 3.3. Factors for Differentiation Between I-AE and iT-AE

**Figure 3:**
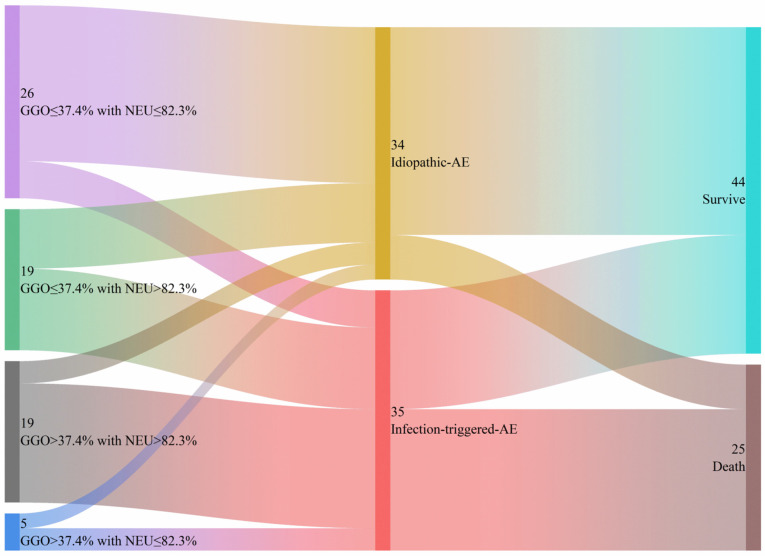
The Sankey graph illustrates the likely clinical course of patients with IIM-ILD during the first year following AE onset, categorized by different causes. AE, acute exacerbation; IIM-ILD, idiopathic inflammatory myopathy-associated interstitial lung disease.

As shown in Table 2, the univariate and multivariate logistic regression analyses revealed that the NEU (odds ratio [OR] = 1.10; 95% confidence interval [CI] = 1.01–1.20; *p* = 0.03) and the extent of GGO on HRCT (OR = 1.53, 95% CI = 1.02–2.30; *p* = 0.041) are significant independent predictors of iT-AE. A combination of NEU and GGO extent could help discriminate I-AE patients from iT-AE patients; the AUC is 0.812 (95%CI : 0.711–0.913), while the sensitivity, specificity, and accuracy are 71.4%, 73.5%, and 72.5%, respectively (Figure 2B). The optimal cut-off values for NEU and GGO extent are 82.3% and 37.4%, respectively. We used a Sankey graph to visually demonstrate the likely clinical trajectory of patients with IIM-ILD in the subsequent year following AE onset according to different etiologies (Figure 3).

## 4. Discussion

The present study revealed that approximately half of AEs in patients with IIM-ILD were triggered by infection, predominantly by viral and fungal pathogens. Patients experiencing iT-AE exhibited more pronounced hyperinflammation and had worse short- and long-term outcomes compared with those with I-AE. Furthermore, a combination of NEU and the GGO extent on HRCT at AE onset may assist clinicians in promptly identifying the underlying AE-ILD etiology and guiding appropriate management strategies.

In our cohort, 50.7% of AEs in patients with IIM-ILD were infection-triggered, with viruses and fungi being the most common pathogens. Importantly, mortality in the iT-AE group was significantly higher than in the I-AE group at every time point evaluated (30-day: 28.6% vs. 5.9%; 90-day: 34.3% vs. 14.9%; 1-year: 54.3% vs. 17.6%), with the Kaplan–Meier analysis confirming poorer cumulative survival. These findings suggest that infection not only precipitates AE but also substantially worsens both the short- and long-term prognosis. Our findings are partly consistent with previous studies. Liang et al. reported that infection accounted for 53.1% of AEs in IIM-ILD, predominantly bacterial and fungal, and showed a strong association between bacterial infection and adverse short-term outcome [13]. Lee et al. observed that 47.2% (17/36) of AEs were infection-related in their IIM-ILD cohort, mainly viral [15], while Bai et al. demonstrated that infection was identified in 48.9% (65/133) of patients with IIM-ILD and was the leading cause of death (49.3%) [26]. However, our study differs in showing a clear survival gap between I-AE and iT-AE groups across multiple time points. By contrast, Lee et al. found no significant difference in short-term mortality (41.2% vs. 52.6%, *p* = 0.724) in a smaller cohort of 36 patients [15]. These discrepancies may reflect differences in cohort size and statistical power, patient characteristics, the infectious pathogen spectrum across cohorts and centers, or heterogeneity in immunosuppressive regimens and antimicrobial therapy. Mechanistically, infection is increasingly recognized as a critical trigger of AE. Evidence from IPF demonstrates that AE is associated with an elevated microbial burden and an altered respiratory microbiota composition compared with those in stable disease [6,7,8]. Such dysbiosis may amplify host immune responses and exacerbate preexisting lung injury, leading to poor survival. Our results, together with prior evidence, underscore the need for rigorous infection prevention, prompt diagnostic evaluation, and tailored management in IIM-ILD. For iT-AE, treatment requires careful balancing of immunosuppression and antimicrobial therapy. While aggressive immunosuppression remains central in I-AE, our data suggest that excessive immunosuppression in iT-AE may worsen outcomes by impairing host defense. Tailored strategies, such as cautious tapering of immunosuppressants, judicious corticosteroid use, and early empiric antimicrobial therapy with subsequent de-escalation, may improve survival. In addition, clinicians should maintain a high index of suspicion for infection when evaluating AE and prioritize early, comprehensive diagnostic work-up. Preventive approaches, including antimicrobial prophylaxis in high-risk patients, and vigilant monitoring during immunosuppressive therapy warrant consideration. Future multi-center, prospective studies with standardized outcome reporting are warranted to validate these findings and to explore strategies aimed at reducing infection-related AE and improving survival in IIM-ILD.

From a pathophysiological perspective, the worse prognosis of iT-AE is biologically plausible. Our study demonstrated distinct clinical and radiological differences between iT-AE and I-AE. Clinically, patients who experience iT-AE presented in a poorer condition, with lower HGB and PaO_2_/FiO_2_ ratios, and exhibited more pronounced systemic inflammation—evidenced by higher pulse, body temperature, WBC count, NEU, and CRP, ESR, LDH, and HBDH levels compared with those in I-AE. These findings may suggest that unlike the intrinsic acceleration of underlying IIMs in I-AE, infection may provoke a more acute, hyperinflammatory response and cause direct, extensive lung tissue injury, leading to worsening ILD, particularly in immunocompromised individuals. These findings are consistent with previous reports. Jang et al. reported lower PaO2/FiO2 ratios and higher LDH levels in infection-triggered AE-ILD compared with those in non-triggered AE-ILD [27]. Similarly, Kato et al. observed higher CRP levels and a lower forced vital capacity (FVC) and vital capacity (VC) in infection-triggered versus idiopathic AE of interstitial pneumonia [28]. However, the precise pathophysiological mechanisms underlying these differences remain to be fully elucidated, and the clinical significance of these individual parameters in isolation is uncertain. Therefore, these indicators should be interpreted within the broader clinical and radiological context, and further studies are needed to confirm their utility in routine practice. Radiologically, patients who experienced iT-AE displayed more extensive GGOs on HRCT than those who experienced I-AE. GGO is an essential characteristic for the definition of AE of ILD. On HRCT, GGO usually represents partial filling or collapse of the airspaces, interstitial thickening, an increased capillary blood volume, or a combination [23]. In patients with AE-ILD, GGO is likely to be associated with pathological DAD, characterized by alveolar and interstitial edema, alveolar fibrinous exudate with hyaline membranes, type II pneumocyte hyperplasia, and increasing inflammatory cells, such as neutrophils and lymphocytes [5]. The finding that patients with iT-AE exhibited more extensive GGO than those with I-AE (which is suggestive of primary IIM activity) suggests that infection may strongly activate the host immune system, triggering a vigorous inflammatory response. This process can drive excessive cytokine release, uncontrolled accumulation of inflammatory cells, and the generation of cytotoxic mediators such as reactive oxygen species, thereby exacerbating lung injury and resulting in more extensive radiological abnormalities [29].

Beyond descriptive findings, our study contributes to diagnostic stratification. Multivariate logistic regression revealed that NEU and GGO extent were independently associated with an increased risk of infection. The combination of NEU and GGO extent facilitated differentiation between iT-AE and I-AE patients, with an AUC of 0.812 (95%CI: 0.711–0.913, sensitivity: 71.4%, specificity: 73.5%). While bronchoscopy with bronchoalveolar lavage remains informative for AE-ILD etiology, its use is often limited by safety concerns [6,30]. Our results introduce a novel, non-invasive, and practical method for differentiating iT-AE from I-AE. Previous studies have demonstrated the prognostic value of radiological and laboratory parameters in AE-ILD. For instance, Zhang et al. highlighted HRCT-based risk signatures in AE of IIM-ILD [22], while Ba et al. identified specific inflammatory markers as independent predictors of outcome [31]. Our finding indicates that integrating radiological and laboratory parameters could enhance prognostic modeling in AE-ILD. Collectively, these results support a combined biomarker–imaging approach as a promising strategy for early diagnosis, risk stratification, and therapeutic decision-making. Early identification of AE-ILD etiology enables personalized treatment, such as intensifying immunosuppression in I-AE or initiating targeted antimicrobial therapy in iT-AE, thereby improving clinical outcomes. Thus, our study adds meaningful evidence to the field and has the potential to advance patient care.

This study has several limitations. First, as a single-center retrospective study with a relatively small sample size, selection bias cannot be excluded, and the generalizability is constrained. Although we consecutively screened all IIM-ILD patients experiencing AE during the study period, inclusion required an available chest HRCT scan. Consequently, patients who died before imaging or were managed elsewhere were excluded, potentially underestimating AE severity and mortality in this population. Nevertheless, our real-world data provide meaningful exploratory insights. Second, bronchoalveolar lavage was infeasible in critically ill patients, risking infection under-detection and I-AE/iT-AE misclassification despite systematic testing. Third, pulmonary function tests were omitted in nearly two-thirds of patients due to intolerance at AE onset, precluding baseline disease severity assessment as a prognostic factor—though prior studies suggest limited prognostic value of AE etiology differentiation in RD-ILD. Fourth, the sample size precluded a robust mortality risk factor analysis through subgroup comparisons. Finally, the retrospective design restricted our ability to establish causal relationships. Future multicenter prospective studies with larger cohorts should validate our findings, identify high-risk patients, and explore prophylactic strategies against opportunistic infections to optimize outcomes.

## 5. Conclusions

In summary, this preliminary study demonstrates that infection is a frequent and clinically important trigger of AE in patients with IIM-ILD. Compared with I-AE, iT-AE is characterized by heightened systemic inflammation, greater radiological involvement, and substantially worse short- and long-term survival. NEU and the GGO extent on HRCT at AE onset showed a moderate ability to distinguish between iT-AE and I-AE. These markers may nevertheless serve as adjunctive indicators to raise clinical suspicion and inform timely management. Future prospective, multi-center studies are warranted to refine the risk stratification and establish evidence-based management strategies for this high-risk population.

## Figures and Tables

**Table 1:** Clinical features of idiopathic and infection-triggered acute exacerbation of IIM-ILD.

Characteristics	Total (*n* = 69)	Idiopathic (*n* = 34)	Infection-Triggered (*n* = 35)	*p* Values
Sex (female), *n* (%)	55(79.7)	25(73.5)	30(85.7)	0.244
Age at AE, y	50.7(10.0)	50.9(9.56)	50.4(10.4)	0.823
BMI(Kg/m^2^)	21.4(3.4)	21.6(3.0)	21.3(3.7)	0.768
SBP (mmHg)	115.8(17.4)	117(15.5)	115(19.2)	0.618
DBP (mmHg)	74.5(9.9)	75.2(10.3)	73.8(9.58)	0.559
Pulse	102(19.7)	96.1(17.5)	108(20.3)	0.013
Body temperature (℃) (M, IQR)	36.7(1.6)	36.5(0.8)	37.5(1.6)	0.037
IIM subtypes, *n* (%)				0.799 *
DM	50(72.5)	24(70.6)	26(74.3)	
PM	11(15.9)	5(14.7)	6(17.1)	
Overlap in IIM	8(11.6)	5(14.7)	3(8.6)	
Autoantibody #, *n* (%)				0.213 *
Anti-ARS	18(26.1)	11(32.4)	7(20.0)	
Anti-Jo-1	13(18.8)	6(17.6)	7(20.0)	
Anti-EJ	1(1.4)	1(2.9)	0(0)	
Anti-PL-7	4(5.8)	4(11.8)	0(0)	
Anti-MDA5	10(14.5)	2(5.9)	8(22.9)	
Anti-Ro52	20(29)	9(26.5)	11(31.4)	
IIM disease duration, m, (M, IQR)	8(15.7)	9(15.9)	8(16.4)	0.564
Smoking, *n* (%)				1.00 *
Ever-smokers	9(13.0)	4(11.8)	5(14.3)	
Never-smokers	60(87.0)	30(88.2)	30(85.7)	
Comorbidity, *n* (%)	55(79.9)	26(76.5)	29(82.9)	0.561
Treatments before AE, *n* (%)				
Maintenance CS therapy	53(76.8)	26(76.4)	27(77.1)	0.947
Cyclophosphamide	21(30.4)	10(29.4)	11(31.4)	0.733
Cyclosporin	4(5.8)	1(2.9)	3(8.6)	1.00 *
Mycophenolate mofetil	7(10.1)	2(5.9)	5(14.3)	0.71 *
Hydroxychloroquine	18(26.1)	10(29.4)	8(22.6)	0.535
Thalidomide	10(14.5)	6(17.6)	4(11.4)	0.513 *
Tofacitinib	2(2.9)	1(2.9)	1(2.9)	1.00 *
Anti-fibrotic therapy	4(5.8)	3(11.7)	1(2.9)	0.356 *
Laboratory data (M, IQR)				
WBCs (10^9^/L)	7.8(5.7)	7.1(5.9)	8.5(6.4)	0.071
NEU	83.1(14.7)	79.2(18.9)	87.6(10.1)	<0.001
HGB (g/dL)	120(18.6)	125(17.4)	115(18.7)	0.029
PLTs (×10^4^/µL)	230.3(96.5)	241(117)	220(71.1)	0.361
CRP (mg/dL)	22(45.0)	10.7(31.8)	30.9(77.9)	0.029
ESR (mm/h)	33(44.5)	28(37.3)	49(48)	0.015
CK(U/L)	79(579.3)	67(734.6)	182(524.5)	0.384
LDH (U/L)	443(331)	353(176.8)	594(402.4)	<0.001
HBDH (U/L)	344(210.5)	289(126.8)	428(338)	<0.001
PaO_2_/FiO_2_ ratio	218.6(94)	249(81.7)	189(96.6)	0.008
HRCT features, %, (M, IQR)				
GGO	27.4(25)	20.6(22.1)	38.1(28.8)	0.001
Reticulation	10(13.4)	10.2(15)	10.1(12.4)	0.838
Honeycombing	0(0)	0(0)	0(0)	0.293
Consolidation	10.6(15.6)	7.8(16.7)	11(14.5)	0.592
Emphysema	0(0)	0(0)	0(0)	0.647
Treatments after AE, *n* (%)				
CS pulse therapy	22(31.9)	12(35.3)	10(28.6)	0.549
Cyclophosphamide	19(27.5)	13(38.2)	6(17.1)	0.05
Cyclosporine	4(5.8)	3(8.8)	1(2.9)	0.356 *
Mycophenolate mofetil	6(8.7)	4(11.8)	2(5.7)	0.428 *
Hydroxychloroquine	10(14.5)	5(14.7)	5(14.3)	1.00 *
Thalidomide	3(4.3)	2(5.9)	1(2.9)	0.538
Tofacitinib	3(4.3)	1(2.9)	2(5.7)	1.00 *
Tocilizumab	2(2.9)	0(0)	2(5.7)	0.493 *
IVIG	25(36.2)	10(29.4)	15(42.9)	0.245
Antimicrobial therapy	65(94.2)	31(91.2)	34(97.1)	0.289
Mechanical ventilation	13(18.8)	4(11.8)	9(25.7)	0.138
Status(death), *n* (%)	25(36.2)	6(17.6)	19(54.3)	0.002

Definition of abbreviations: y, year; m, month; IIM, idiopathic inflammatory myopathy; ILD, interstitial lung disease; BMI, body mass index; SBP, systolic blood pressure; DBP, diastolic blood pressure; DM, dermatomyositis; PM, polymyositis; Anti-ARS, anti-aminoacyl-tRNA synthetase antibody; Anti-MDA-5, anti-melanoma differentiation-associated gene 5 antibody; CS, corticosteroid; WBC, white blood cell; NEU, neutrophil percentage; HGB, hemoglobin; PLTs, platelets; CRP, C-reactive protein; ESR, erythrocyte sedimentation rate; CK, creatine kinase; LDH, lactate dehydrogenase; HBDH, hydroxybutyrate dehydrogenase; HRCT, high-resolution computed tomography; GGO, ground-glass opacity; IVIG, intravenous immunoglobulin. #: Forty-eight patients with autoantibody profiles were assessed. *: Fisher’s exact test.

**Table 2:** Factors selected for distinguishing between idiopathic and infection-triggered acute exacerbation of IIM-ILD according to univariate and multivariate logistic regression analyses.

Variates	Per Unit	Univariate Analysis			Multivariate Analysis		
		OR	95% CI	*p* Value	OR	95% CI	*p* Value
HGB	1 g/dL	0.97	0.94–1.00	0.035	0.97	0.93–1.02	0.223
NEU	1%	1.11	1.04–1.18	0.001	1.10	1.01–1.20	0.03
ESR	1 mm/h	1.02	1.00–1.04	0.024	1.00	0.97–1.03	0.861
LDH	1 U/L	1.00	1.00–1.01	0.002	1.01	0.97–1.01	0.265
HDBH	1 U/L	1.01	1.00–1.01	0.002	1.00	0.98–1.01	0.551
PaO_2_/FiO_2_	1 mmHg	0.99	0.99–1.00	0.01	1.00	1.00–1.01	0.769
GGO	10%	1.59	1.17–2.16	0.003	1.53	1.02–2.30	0.041

Definition of abbreviations: IIM, idiopathic inflammatory myopathy; ILD, interstitial lung disease; CI, confidence interval; OR, odds ratio, HGB, hemoglobin; NEU, neutrophil percentage; ESR, erythrocyte sedimentation rate; LDH, lactate dehydrogenase; HBDH, hydroxybutyrate dehydrogenase; GGO, ground-glass opacity.

## Data Availability

The original contributions presented in this study are included in the article. Further inquiries can be directed to the corresponding author.

## Abbreviations

The following abbreviations are used in this manuscript:

AE	Acute exacerbation
IIMs	idiopathic inflammatory myopathies
ILD	Interstitial lung disease
HRCT	High-resolution computed tomography
GGO	Ground glass opacity
NEU	Neutrophil percentage

## Appendix A

**Table A1:** Interobserver agreement and correlation for the average extent score of each HRCT finding.

HRCT Findings	Average Extents ^a^	Interobserver Agreement ^d^		Interobserver Correlation ^e^	
		ICC Value	*p* Value	Spearman r	*p* Value
GGO	27.37 [18.75, 43.17]	0.986	<0.001	0.978	<0.001
Reticulation	10.08 [4.67, 18.00]	0.959	<0.001	0.948	<0.001
Honeycombing ^b^	0.00 [0.00, 0.00]	0.996	<0.001	1.000	<0.001
Consolidation	10.62 [2.08, 17.50]	0.986	<0.001	0.986	<0.001
Emphysema ^c^	0.00 [0.00, 0.00]	0.911	<0.001	1.000	<0.001

Abbreviations: GGO, ground-glass opacity. ^a^ Average overall scores (mean of the data from two observer groups) are expressed a median percentage of lung parenchyma and the 25th to 75th percentile of the interquartile ranges in the parentheses. ^b, c^ There were only four and thirteen cases with positive findings in honeycombing and emphysema, respectively. The mean values were 0.47 (range, 0.67–19.33) and 0.32 (range, 0.13–7.75) in honeycombing and emphysema, respectively. ^d^ Interobserver agreements between two observers were analyzed using ICC statistics (ICC value) ^e^ Interobserver correlations between two observers were analyzed using Spearman’s rank correlation test (*r* value).
